# Edge Computing to Secure IoT Data Ownership and Trade with the Ethereum Blockchain

**DOI:** 10.3390/s20143965

**Published:** 2020-07-16

**Authors:** Anum Nawaz, Jorge Peña Queralta, Jixin Guan, Muhammad Awais, Tuan Nguyen Gia, Ali Kashif Bashir, Haibin Kan, Tomi Westerlund

**Affiliations:** 1 Shanghai Key Laboratory of Intelligent Information Processing, School of Computer Science, Fudan University, Shanghai 200433, China; 18110720163@fudan.edu.cn (A.N.); 17210240095@fudan.edu.cn (J.G.); 2 Turku Intelligent Embedded and Robotic Systems Group (TIERS), Faculty of Science and Engineering, University of Turku, FI-20014 Turku, Finland; jopequ@utu.fi (J.P.Q.); tunggi@utu.fi (T.N.G.); tovewe@utu.fi (T.W.); 3 School of Information Science and Engineering, Fudan Univeristy, Shanghai 200433, China; 17110720061@fudan.edu.cn; 4 Department of Computing and Mathematics, Manchester Metropolitan University, Manchester M15 6BH, UK; dr.alikashif.b@ieee.org; 5 Fudan-Zhongan Joint Laboratory of Blockchain and Information Security, Shanghai Engineering Research Center of Blockchain, Shanghai 200433, China

**Keywords:** IoT, blockchain, edge computing, data ownership, data trade, information security

## Abstract

With an increasing penetration of ubiquitous connectivity, the amount of data describing the actions of end-users has been increasing dramatically, both within the domain of the Internet of Things (IoT) and other smart devices. This has led to more awareness of users in terms of protecting personal data. Within the IoT, there is a growing number of peer-to-peer (P2P) transactions, increasing the exposure to security vulnerabilities, and the risk of cyberattacks. Blockchain technology has been explored as middleware in P2P transactions, but existing solutions have mainly focused on providing a safe environment for data trade without considering potential changes in interaction topologies. we present EdgeBoT, a proof-of-concept smart contracts based platform for the IoT built on top of the ethereum blockchain. With the Blockchain of Things (BoT) at the edge of the network, EdgeBoT enables a wider variety of interaction topologies between nodes in the network and external services while guaranteeing ownership of data and end users’ privacy. in EdgeBoT, edge devices trade their data directly with third parties and without the need of intermediaries. This opens the door to new interaction modalities, in which data producers at the edge grant access to batches of their data to different third parties. Leveraging the immutability properties of blockchains, together with the distributed nature of smart contracts, data owners can audit and are aware of all transactions that have occurred with their data. we report initial results demonstrating the potential of EdgeBoT within the IoT. we show that integrating our solutions on top of existing IoT systems has a relatively small footprint in terms of computational resource usage, but a significant impact on the protection of data ownership and management of data trade.

## 1. Introduction

A wider adoption of the Internet of Things (IoT) devices and systems across different industries and domains come together with an exponential increase in the amount of real-time data being generated and processed) [1]. From the point of view of data privacy and trade between devices at the edge acquiring the data and cloud-based third-parties processing it, multiple challenges remain [2]. While the IoT emerged with the adoption of cloud technology, in recent years more distributed approaches are being adopted and new paradigms are emerging [3]. Among these tendencies, the edge computing paradigm has materialized as those architectures that shift most of the data processing closer to where it is being generated, at the edge of the network. This shift and optimization in computation also lead to better network usage and significant reductions in the network load. However, the change in network topologies also comes with a new set of data privacy and security implications that must be considered [4]. Blockchain technology provides inherent security, proof of ownership, and identification, hence being a potential solution for many of the cybersecurity challenges in the IoT [5]. By taking advantage of the built-in consensus mechanisms, IoT systems can rely on blockchain for managing data integrity and immutability [6].

A recent trend in the IoT is to distribute the data processing and analysis through a series of network layers, extending a traditional cloud-centric architecture [7]. By shifting the computation load towards the edge of the network, applications can benefit from advantages, including lower latency and more optimized network load [8]. Furthermore, in many applications, raw data do not need to be stored and only the result of its analysis is kept [9]. Therefore, it is no longer the best strategy to continuously transmit real-time data to the cloud. In turn, embracing the edge computing paradigm reduces the possibility of data privacy violations [10], but it also raises additional security considerations, including reliability of information and authenticity of the data sources. In some scenarios, such as health-care IoT, edge computing can be leveraged for increasing the security of personal data [11]. Nonetheless, the addition of intermediate layers between IoT devices, cloud servers, and end-users applications increases the exposure to security flaws, injection of malicious code, and cyberattacks [12].

Over the past decade, an amalgamate of IoT products, solutions, and systems have been penetrating both industrial and domestic domains, which has led to a growing number and variety of security vulnerabilities and cyberattacks [13]. Proper security methods gain special significance in environments where personal data is being gathered, such as smart homes [14]. In particular, voice assistants are becoming increasingly accessible in the consumer electronics market, creating a direct risk to end-users privacy [15]. The utilization of current encryption methods is not enough to protect users’ data and privacy [16]. Therefore, the need for a more robust solution for sharing and trading personal data securely and safeguarding privacy is evident.

Since the introduction of Bitcoin in 2008, blockchain technology has become increasingly utilized in various domains, mostly for finance-related applications, or as an immutable distributed data storage solution [17]. it was with the introduction of ethereum and the ability of running short programs within the blockchain, when a vast number of applications emerged, in particular in the IoT field [18]. With proof of ownership and distributed data transactions, blockchain technology provides a natural channel for trade between data producers or sellers (edge devices) and data consumers or buyers (i.e., third-party applications) [19]. Current works integrating blockchain for the IoT focuses on securing individual transactions between applications and edge devices [20], or among devices [21]. We believe that its potential at a system level for integrating edge devices as data producers and applications or third-party services as data consumers is yet to be explored in more detail. Therefore, we put a focus on the problem of data ownership, enabling the integration, validation, control, and audit of third party access to IoT data.

This paper introduces EdgeBoT, an IoT system architecture exploiting edge computing for data processing and the ethereum blockchain as a distributed middleware. EdgeBoT enables direct peer-to-peer data transactions in the Blockchain of Things (BoT). Our proposed model transfer the data trading rights directly to their producers (edge computing devices), which rely on smart gateways to run the blockchain. In particular, we are interested in the ethereum blockchain because of the possibility to run smart contracts, scripts that are validated as part of a transaction in the blockchain. Smart contracts are written in a scripting language designed as part of ethereum and executed and validated in a decentralized manner validation of [22]. We leverage the inherent secure nature of these contracts to secure and control third-party access to IoT data. Furthermore, the immutability of a blockchain transactions’ records permits the audit of past transactions by any node in the network.

The rest of this paper is organized, as follows. Section 2 explores related works in the use of blockchain technology in IoT platforms. Section 3 introduces the EdgeBoT architecture and presents its advantages for secure integration of third-party services with IoT devices. In Section 4, Experimental data, results, and validation of EdgeBoT are presented in Section. Finally, Section 5 concludes the work and describes future research directions.

## 2. Related Work

Although considerable research has been conducted on security and privacy solutions for IoT devices [14,23,24,25], edge-based solutions for data security were also presented frequently in recent years [26,27,28], and specifically distributed blockchain-based solutions for data privacy. Since its early development, blockchain technology has been seen as a candidate to solve many of the security challenges that centralized data exchange models inherently have, due to its decentralized nature and full end-to-end encryption scheme. Table 1 lists out major challenges and problems in the existing centralized infrastructure and their proposed solutions based on blockchain [18,29,30]. In this section, we review and summarize previous works on exploiting blockchain-based solutions to increment data privacy, data ownership, and secure P2P transfer data within the IoT.

In [31], Li et al. propose an efficient and secure mobile healthcare system, Edgecare, which provides a hierarchical distributed architecture to manage and guarantee healthcare data privacy by leveraging edge computing with stackelberg game-based optimization algorithm to achieve fair data trading. In another study [32], Lin et al. present Edge-AI enabled architecture to make sensory data trade-able as a knowledge. Authors work on a proof-of-trade consensus mechanism and non-cooperative game based optimum knowledge approach to build a knowledge market. In a recent review by Krittanawong et al.  [33] summarizes the potential opportunities and challenges by integrating blockchain with AI to develop personalized medicine for cardiovascular, according to their review this combination can work as a booster for reliable data availability needed for personalized medicines, but still, it is needed to consider the privacy of patients and data producers. Debe et al. [34] proposed an IoT based decentralized trust model that consists of fog nodes to get the reputation of publicly available fog nodes. Trust level is retained by using the feedback from existing users and interactions, by using this approach reputation management system will become more reliable and transparent as compared to existing third party based systems. In another recent research work [35], Mazzei et al. proposed and implemented a trustless industrial solution that acts as a bridge between IoT solutions and the virtual world of digital twins. This proposed blockchain-based solution provides a standalone interoperable tracking system for industrial applications. When compared to this and other solutions available, our proposed architecture is so far generic, and we have focused towards discussing a system-level view rather than on specific integrations, which will be the objective of our future works.

Yuan et al. [36] designed and implemented an emission trading system that is based on hyperledger, another open-source permissioned blockchain-based distributed system. This system can be leveraged for increasing credibility in trading services for polluters by sharing immutable transactions in a chain. In another study by Rehman et al. [37], a quite similar approach is used for sharing economy services. Cognitive edge framework that is based on blockchain technology is proposed to secure smart city services by using smart contracts and off-chain nodes to store immutable records. AI is used to extract informative information from existing records for training datasets need to use for smart contract logic. In addition to academia, Popov et al. [38] proposed *iota*, a distributed ledger for commercial IoT products and services. When compared to a traditional blockchain architecture, iota replaces the chain for the tangle, a data structure based on a directed acyclic graph that enables better scalability and lower latency for transaction confirmation [39]. Iota solves the main challenge preventing micro-payments being more widely adopted with blockchain in the IoT: the fact that the cost of processing a transaction is higher than the amount of currency involved in the transaction itself. In iota, there are no transaction fees. This is possible, because, in order to make a transaction, nodes need to validate two other transactions. This scenario is quite relevant to the approach we implement in our model. In EdgeBoT, a node does not need to compute existing transactions to save new transactions in the chain while in *iota*, the node first needs to participate in the validation of two other transactions.

In a comprehensive survey, Yang et al. [40] addressed significant challenges, including security and privacy, self organization, functions integration, scalability, and resource management of integrated blockchain based edge systems. The authors also proposed a comprehensive classification of state of the art solutions by providing use-case based scenarios and attempts to explore the technologies working near the blockchain-based edge computing domain. Cha et al. [41] proposed a robust digital signature scheme for blockchain connected gateway to maintain user privacy preferences for IoT devices securely. Bergquist et al. [42] proposed and implemented blockchain technology and smart contracts as means of building privacy-sensitive applications. The authors used medication plans as a primary use-case but they proposed that results can be shifted to other applications where sensitive data are being shared and a proof of legitimacy or authentication is required.

Another significant challenge of edge computing based on blockchain is scalability. Instead of chain models, researchers come up with different solutions. Poon et al. [43] come with a scalable decentralized autonomous blockchain solution Plasma based on edge computing, which can update billion state updates within a second. They propose blockchain consortium in a MapReduce format, which allows for child chains (sidechains) over a hierarchical tree to come up with the scalability problem. It allows efficient matching of hierarchical ledger topology and provides fixed withdrawal delays to increase its scalability by minimizing the damage. It works in asynchronous mode during transaction handling. A similar approach was proposed by the Zilliqa team [44], where they use the concept of sharding to cope with scalability issues. In this work, they divided the load into smaller shards that can process parallel transactions. Along with sharding, a new scripting language for special propose smart contracts and execution environment as an under-laying architecture was proposed. Which makes a considerable difference in a computation platform for high scale parallel transactions. Another similar work is done by Sompolinsky et al. [45], who proposed a tree-based distributed ledger instead of chain framework. They focused on network delay effects, which lead to double-spend attacks. The GHOST protocol is introduced by modifying the bitcoin protocol, which increases security by reducing the confirmation time of transaction.

Most of the existing applications were focused on securing connectivity between end-users and specific IoT devices, routing connections through a blockchain-based network. However, to the extent of the authors’ knowledge, little to no attention has been put in exploiting blockchain and smart contracts to provide P2P data trade and data ownership rights for data-sensitive environments to its’ producers. Therefore, we propose an EdgeBoT platform that adapts to a wide variety of M2M interaction topologies. At the same time, it is scalable and takes into account the integration of both smart gateways and end-devices at the edge of the network, with a wide range of computing capabilities.

## 3. EdgeBoT: P2P Data Trade Architecture

Transactions that involve data exchanges strongly rely on third-parties that act as intermediaries. Traditionally, data acquired by end-devices has been analyzed, aggregated and stored by cloud services. Third-party cloud services, in turn, act as a bridge between data producers and consumers. The strong dependency of the digital world on the third-party cloud services opens the door to a wide variety of vulnerabilities in terms of security and data privacy protection. More recently, the rising paradigm of fog/edge computing has broadened the network stack by adding extra layers between end-devices and cloud services or other third parties. These layers, which are closer to both data consumers and producers, are often less secure than cloud services and increase the number of possible vulnerabilities in the overall system [46]. While blockchain was originally designed for data storage security, immutability, and auditability, it also has the advantage of allowing end-users or devices to exchange digital assets directly without any intermediate third parties involved in the process [29,47].

EdgeBoT is a network architecture that consists of manager nodes (arbitrator, regulatory authorities, handlers), edge gateways, sensors, and actuators, as well as end-user applications (data buyers). The overall system divides into the following layers:

1. Sensor layer includes individual sensors, actuators or other light nodes which do not possess any computational or storage capabilities to participate in a network for data processing and trade.
2. Edge layer consists of a local network (BLE, radio access points) and single-board computers (SBC).
3. Fog layer consists of manager nodes which work as an arbitrator, regulatory authorities, transaction and data handlers for the Ethereum blockchain.
4. Cloud layer provides a place for applications and storage.

**Figure 1:**
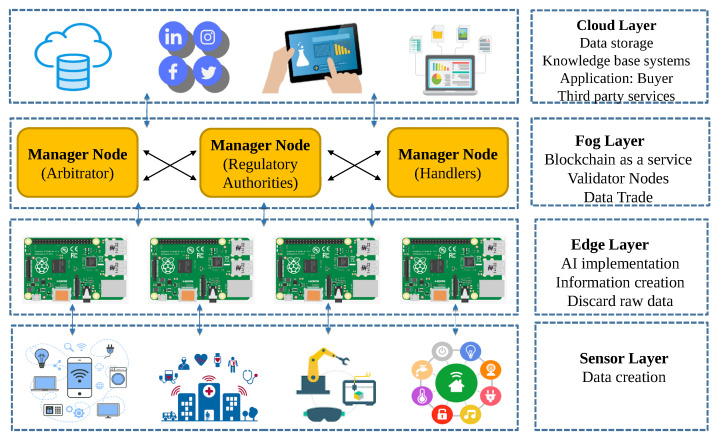
Architecture of EdgeBoT, a distributed P2P data trade and fair access network model based on the ethereum platform.

**Figure 2:**
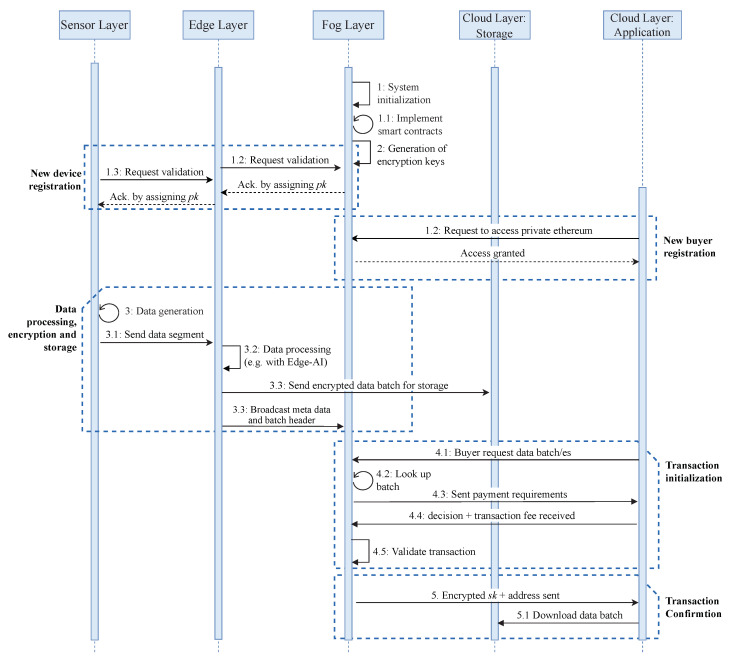
System sequence diagram depicting sequential picture of complete network.

Figure 1 illustrates the architecture of the deployed EdgeBoT network, with P2P distributed communication and data exchange. Apart from cloud services and applications running on other powerful computing platforms, manager nodes at the Fog layer represent the bulk of the network nodes that work independently in a fully autonomous way (pre-defined smart contracts or scripts). These require higher computational capabilities to handle a large number of parallel operations. The Fog layer is directly connected to the Cloud layer providing the final step in forming the final link between the Sensor and Cloud layers. The Fog layer is responsible to initialize a network, the System initialization process in the sequence diagram that is shown in Figure 2. The Fog layer is also fully responsible for running the Ethereum network and creating smart contracts. Therefore, edge gateways at the Edge layer first request to join the private Ethereum network (the New device registration block in Figure 2) to obtain the cryptographic keys generated at the Fog layer. By private we do not mean a consortium blockchain where certain nodes have higher authority, but only that new nodes will need to request access to a single manager for joining the blockchain (after which the manager is just a normal node). Edge gateways can connect and handle several sensors and actuators located at the Sensor layer. Sensors are responsible to gather data and send them to the upper layer for processing and storage, the Data processing, encryption and storage block Figure 2. Whenever a new node wants to buy or sell data in the EdgeBoT network, it first needs to register in the network, the New buyer registration block in Figure 2. During this operation, a child key derivation (CKD) function generates both private-public keys for the node. At that point, the buyer node becomes a full member of the network by creating its public key derived from network key and has access to the encrypted history of transactions and all previous data batches that have been generated since the establishment of the platform.

### 3.1. Data Processing and Aggregation at the Edge

Edge gateways are smart gateways at the edge of every sidechain and work with local networks only. Edge gateways consist of SBC’s, such as Raspberry Pi boards or Intel UP boards, which have enough computational capabilities to run AI algorithms that are designed for resource-constrained devices. The sensor layer consists of several actuators or sensors which do not possess any computational capabilities to participate directly in the blockchain network. Therefore, sensor nodes rely on the edge gateway that they are connected to, which acts as an intermediary for them. These edge gateways run sidechains to store data hashes of their previous data batches and transaction validation records. Complete flow of generation of data batch from the data segment, its processing at edge gateways, and storage on cloud layer are described in the data processing, encryption, and storage block of sequence diagram shown in Figure 2.

Sensor nodes are connected to one edge node only, which minimizes the vulnerable channels in which data could be compromised. If one sensor node is compromised, an adversary can only affect that particular edge node. Therefore, single encrypted connection significantly increases the levels of data security, ensures fair access to data and proper ownership, adding the sensor nodes to the backbone chain of the EdgeBoT network. While the manager nodes at the Fog layer can communicate with any other manager node in the network in a completely autonomous way, without any external supervision to its actions or reactions. These nodes can also be used as a custom access control model.

### 3.2. Data Trade through Ethereum

The backbone of EdgeBoT is the Ethereum blockchain, which records all transactions that occur within the network by using a time-stamp system and cryptographic hashes to prevent alteration retroactively [48]. By using smart contracts, all of the specifications of any particular user, such as limited access time to a network, can be defined, ensured, and recorded.

**Figure 3:**
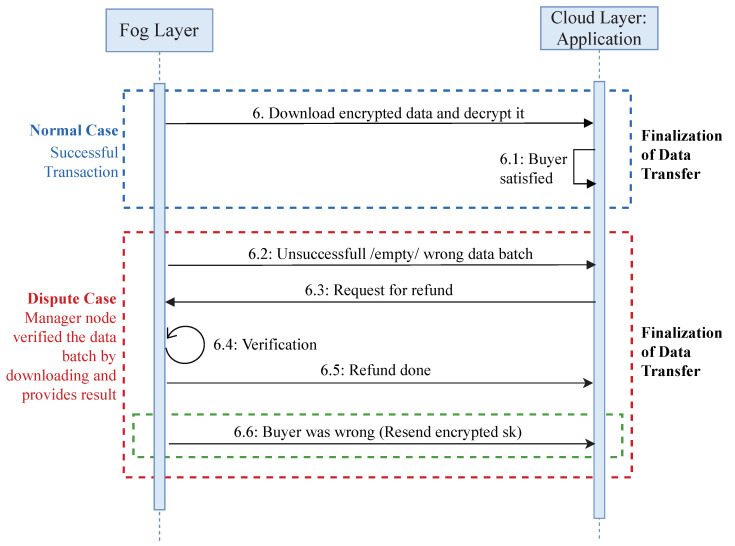
Sequential diagram to elaborate possible scenarios to settle down transaction request.

The complete sequence of data transaction in EdgeBoT is illustrated in the transaction initialization block in Figure 2. Whenever an external buyer (a cloud layer application) wants to acquire data, it joins the network and requests a given data batch while using batch headers that are available publicly on the network. A manager node at the Fog layer replies with the transaction price and conditions. If the buyer accepts the conditions, the manager node validates the transaction and sends the unique secret encryption key (derived from CKD function) of the requested data batch by encrypting it with ECDSA, using the buyer’s public key along with the address of the data batch. The buyer downloads the encrypted data batch from cloud layer (storage) and decrypts it using its public key. After getting the required data, possible scenarios occur which are shown in Figure 3. In a normal case, a buyer is satisfied with the received data. In a dispute case, a buyer is dissatisfied with the received data, or even unable to download or decrypt the data. In this case, the buyer can request for a refund. After getting the refund application, a manager node verifies the request by downloading the required data batch. If the buyer’s request is legitimate, the refund request will be processed. If the buyer was wrong, the manager node sends the encrypted *sk* again, which finalize the transaction request.

### 3.3. Security Measures

The distributed structure of edge/fog computing brings security and privacy challenges for the system involve heterogeneous edge nodes. Several vital security challenges have been identified in [49,50,51]. Distributed Denial of service (DDoS) and Man in the Middle (MITM) different security measure have been taken into account to overcome these reported attack vulnerabilities like Sybil attacks, which is explained in Table 2. DDoS and Time-delay attacks are handled in our proposed system by limiting the number of requests by the edge node to the sensor node and vice-versa. By using different encryption schemes and system elements, EdgeBoT is resilient enough for network and data security attacks. Elliptic curve integrated encryption scheme (ECIES) [52] has been used to save data securely by using the child key derivation function (CKD) [53] for every data batch, the elliptic curve digital signature algorithm (ECDSA) [54] is used to share the unique key of data and communication. To increase security, double authentication of nodes is required after three frequent requests per minute by the same buyer. When a buyer requests data from a manager node that is stored encrypted in a third-party service, i.e., cloud storage. The Manager node sends the encryption key of the requested data batch with its storage location on the cloud is to the buyer instead of sending the requested data batch itself. This scheme is chosen to save bandwidth and computational resources in edge nodes, as well as avoiding storage limitations at the edge. To transfer child secret key *csk*, in a secure way to the buyer, asymmetric encryption is used. Suppose that $csk_{N}$ is the key to the requested data batch *N* of data. Subsequently, the node encrypts $csk_{N}$ with the buyer’s public key, which will be, in turn, decrypted at the receiving end with the buyer’s secret key. Information regarding the location of the stored data is also included in the encrypted payload, together with $csk_{N}$, if that information is not known by the buyer beforehand.

- ECIES is used in key encapsulation mechanism which combined with a data encapsulation mechanism, to encrypt data batch by using the public key of edge node and send encrypted data to managerial nodes. As compared to the more widely used Rivest–Shamir–Adleman (RSA) cryptosystem, elliptic curve cryptography (ECC) requires shorter keys to provide the same levels of security. At the same time, ECC usually requires lower computational and memory resources, which makes it stand strong for scarce computing devices. It is used in practice to establish a key in situations where data transfer is unidirectional or data is stored encrypted under the public key.
- CKD functions are used by hierarchical deterministic wallets to derive children keys from parent keys. This methodology is employed to generate a unique secret key for each data batch in the device to be encrypted. Based on the parent’s public key (private and public keys are both 256 bits), its chain code and the desired child index, and  512-bit hash is generated. The one-way hash function used in the process makes it impossible to obtain the original parent key from the nth-child key. The additions *modulo n* in the process generate seemingly random numbers.
- ECDSA is the first successful standard algorithm based on elliptic curve cryptography, which gained the attention of security researchers due to its robust mathematical structure and smaller key sizes for constrained resource devices. it is used in the communication of buyers and sellers as well as to send unique child secret of required data batch.

Advanced encryption scheme (AES) is used in EdgeBoT to securely store private data, cloud services or other third-party storage solutions. To store the data in a secure, encrypted way is of particular importance to edge nodes or those nodes in the network that do not have enough resources for local storage. AES encryption is chosen due to its high speed and less number of bits required for the encrypted payload as compared to other data encryption standards, like DES. Besides, most newly developed micro-controllers provide hardware modules for AES encryption.

## 4. Implementation and Results

To test the feasibility and reliability of the EdgeBoT architecture in real-life scenarios, the proposed architecture is implemented  using the Raspberry Pi 3 Model B+ minicomputer as an edge gateways. For the implementation of the different parts of the system, the Go programming language (golang), Solidity for the smart contracts, and a suite of web technologies (Node.js^®^, HTML5, CSS3, jQuery) for the front-end application are used. We run Raspbian based on Linux kernel version 4.14.52-v7+ in the Raspberry Pi, which has 1GB of RAM and 4-core ARM processor (BCM2837 @ 1.4GHz). A local Wi-Fi network has been used to enable communication between different edge gateways and its sensor nodes. The cloud servers are simulated with a desktop computer, equipped with an Intel Core i5 processor and 8GB of RAM memory. Remix IDE is used to deploy smart contracts. Data requests and transactions to and from third-party cloud services are generated with Metamask, a browser extension enabling us to initiate parallel transactions during the experiments.

### 4.1. System Implementation

This solution is divided into six processes, as described below. Each process is accompanied by a short pseudo-code algorithm, which describes the different steps. SC is used to refer to smart contracts.

1. System initialization: the generation of encryption parameters, creation of genesis file, and the initialization of the Ethereum blockchain. Define terms of usage, certificates, and policies through smart contracts. A new device/buyer registration process is done through the implementation of smart contracts.
2. Generation of encryption keys: Each connected device generates its key pair of secret and public keys $\left(s_{k},p_{k}\right)$. The secret key is randomly generated, and then the private key and child secret keys are derived from it. Each child secret key is used to encrypt one data batch.
**Process 1:**
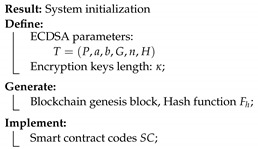

**Process 2:**
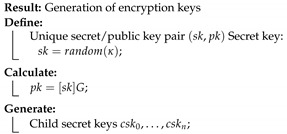
3. Data processing, encryption and storage: Edge gateways divide the acquired data segments into data batches after every time *T* and implement embedded edge AI algorithms. If edge devices do not have enough resources to process data, data is saved as raw data. After encryption, devices call the smart contract function. NewDataBatch to add a transaction to the blockchain. The transaction includes the data hash, the encrypted AES encryption key, the time-stamp of the storage operation, type, and the size of stored data and price. After encryption and broadcasting the hash and metadata, each batch is sent to a storage.
4. Transaction Initialization: When the Application layer (buyer) requests a data batch from manager nodes (seller), it first looks into the available records calling the smart contract function LookUpBatch. If it finds suitable data and its price is within predefined limits, then it initializes a data trade by depositing the amount. This is done calling the smart contract function Deposit, to which a buyer adds a price, its public encryption key, its address or identifier, and the ID of the requested batch.
**Process 3:**
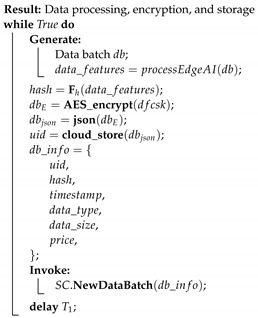

**Process 4:**
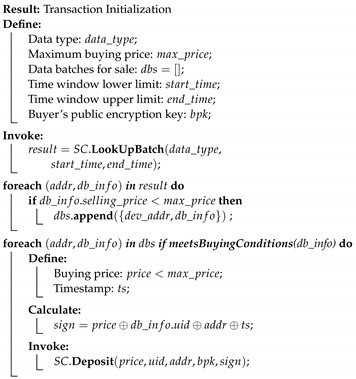
5. Transaction Confirmation: Manager nodes run this process periodically, querying available DataBatch requests with a deposit. If any deposit is found meeting the selling conditions in the requested batch, it validates the transaction, and then it encrypts the csk of the requested batch with the buyer’s public key and confirms the Deal.
6. Finalization of data transfer: When a buyer receives an encrypted data batch AES key, it uses the ECIES decryption algorithm to obtain the AES batch key. Subsequently, it queries the storage provider with the batch address and decrypts it to obtain the information. The data transfer, or purchase, is done if a buyer is satisfied. If the buyer is not satisfied with the received data batch, it will ask for a refund. The manager node will respond to a dispute case by cross-checking the batch details. After obtaining the results, a manager node will respond according to the results.
**Process 5:**
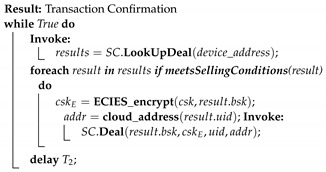

**Process 6:**
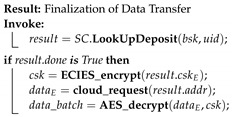

### 4.2. Performance Analyses

**Figure 4:**
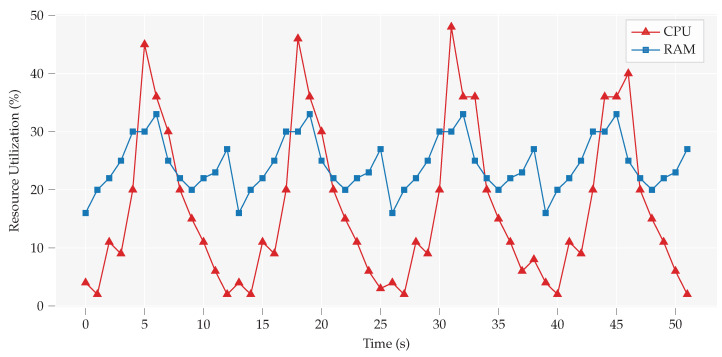
Consumption of CPU and RAM during randomly selected transactions and idle time in between. During transaction handling, CPU requires more resources while in the idle condition it’s quite low. For the RAM, it is not a huge difference during the transaction handling.

Resource consumption has been analyzed at edge gateways to check the required resources to handle the transaction validation. Figure 4 shows the percentage usage of RAM and CPU along *Y*-axis. These results are calculated during transaction validation as well as in an idle condition. In the idle condition, the percentage usage of RAM is 17% as an average, and during transaction, an increase of 10% to 15% is shown. Overall, on average, it used about 26% of RAM resources whose impact on overall system memory usage is not significant during a transaction. However, sharp peaks can be seen in the CPU utilization; in the Idle condition, only 8% CPU resources were used as average, while during a transaction it used up-to 45% of the total. The CPU variance at idle time was of about 10% and about 5% at peak usage.

**Figure 5:**
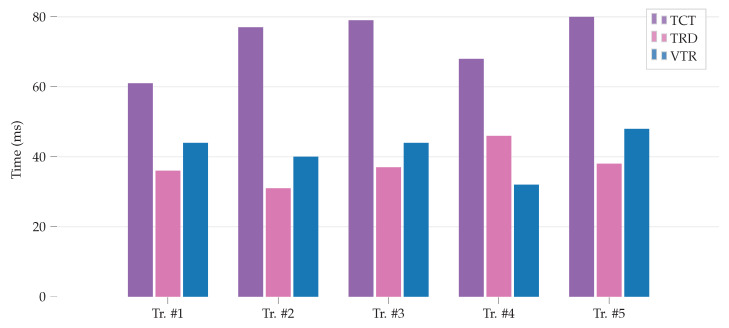
Latencies to retrieve mata data (TRD), transaction validation time (VTR) needed for single transaction request, and time required to confirm one transaction (TCT).

The time that is required to complete a transaction by the edge gateway is measured and subdivided into sections: time required to retrieve metadata (TRD), transaction validation time (VTR), and transaction confirmation time (TCT). The measurements are shown in Figure 5. When considering the available limited resources, it is promising that TRD needs only 34.6 ms on average, VTR 36 ms and TCT 73.6 ms on average. it is worthwhile to note that TCT also relies on the network, which, in this experiment, was affected by our shared Wi-FI network’s slow network response time resulting in increased overall time.

**Figure 6:**
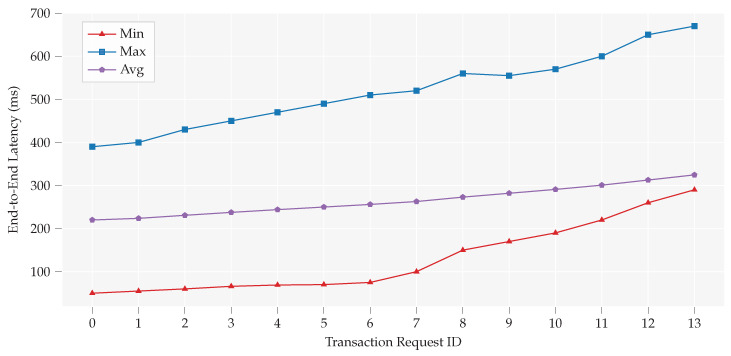
Impact of concurrent transactions on end-to-end transaction latency.

Our second group of experiments focused on the end-to-end delay of concurrent requests: end-to-end delay = request initialization by interested buyer + time to retrieve metadata + response time by manager nodes + time to confirm one transaction. The results in Figure 6 show an increase in the end-to-end delay by the increasing number of concurrent transaction requests. These results from a group of experiments show the efficiency of the proposed model to implement in information critical systems data trade autonomously. This proposed trust-less structure increases reliability and transparency in data trade. it led us to consider that single board computers can work as manager nodes to handle their associated data and transactions without the need for third party cloud services in the future, since the computational resources required leaving room for other edge services and data processing processes to run at the same time. However, increased delay with a parallel number of transactions shows its limitations to be used in mission critical systems where time span comes at first priority.

### 4.3. Scalability Analysis

Edge/fog computing comes with significant benefits; however, it has an inherent scalability barrier that limits its ability to support a wider range of applications. Specifically, system models require robust block creations, involve millions of edge nodes, and a large number of transactions per second. Several researchers have directed their efforts towards mitigating this issue, with different models to replace chain models. Some of the existing proposals that address this challenge are pegged sidechains [55], hierarchical trees [43], cross-chains [56], and DAG [57]. However, in EdgeBoT, scalability enhancement is not a concerning issue, as it is working in a private P2P network that can subdivide this network as sidechains. Edge gateways are not required to process several requests per minute. After every time T, manager nodes need to check whether there are new requests for data. If it has a request in a queue, it will respond to every request one by one.

## 5. Conclusions and Future Work

With the increasing usage of connected devices, the Internet of Things (IoT) is generating a vast amount of data, but the protection of personal data, the online privacy of users and organizations is becoming an increasingly challenging task. Intermediaries and dependence on third-parties act as adversaries and opens the door to a wide variety of vulnerabilities.

In recent years, the edge-computing paradigm, together with ethereum, a distributed ledger, has shown great potential to be widely leveraged and adopted for securing IoT applications. We have presented EdgeBoT, a topology for edge computing (actuators, sensors, and end-user applications) that provides P2P data trade without the need for a third party, making clear the vision of data ownership model. It enables the extension of a private ethereum blockchain to resource-constrained edge devices through a hybrid edge-cloud computing architecture that relies on smart gateways to run the blockchain. EdgeBoT is easily adaptable, scalable, and able to accommodate a wide range of applications.

In EdgeBoT, devices are fully autonomous in terms of their P2P interaction topologies, being able to directly trade their data with third parties. All policies of data trade and ownership rights are implemented through smart contracts. Sidechains are used for parallel transactions and they make a system scalable. They also limit the use of bandwidth and energy needed by edge gateways to update blockchain. To securely store data, devices use elliptic curve integrated encryption scheme (ECIES), generating a unique key for every saved data batch by using child key derivation function (CKD), and double authentication for devices sending requests frequently, which makes our solution resilient enough to vulnerable attacks. To share these unique keys, we have implemented an elliptic curve digital signature algorithm (ECDSA) for communication. The proposed EdgeBot architecture serves as a generic model to secure IoT data ownership rights while preserving the privacy of users. It can be implemented in smart home devices, the industrial internet of things (IIoT), smart health applications (including precision medicine, clinical trials, or accuracy diagnosis), knowledge based systems, or in the research and development of different IoT products.

We executed extensive proof-of-concept experiments to evaluate the performance and reliability of the proposed model. The experiments showed that less than 40% of computing resources were used on average. This indicated that our model can be implemented on various low power single board minicomputers available as off-the-shelf products. Promising results of performance analysis lead us to consider the EdgeBoT as a feasible model for edge computing to secure IoT data ownership and trade.

At this stage, our system shows some limitations of response time, which makes it limited to static environments only. Scenarios where a user needs a rapid response in dynamic environments need further study and will be one of the objectives of our next works. Furthermore, we are exploring a tighter integration of AI algorithms towards embedded and secure edge intelligence and extending our other works in this area [10,58]. we aim at defining new frameworks able to deal with heterogeneous data sources and a wide array of application scenarios.

## Figures and Tables

**Table 1:** Major challenges and problems in existing centralized infrastructure and description of how blockchain aids in overcoming these challenges. The challenges are summarized from the related works listed in this paper, mainly from [18,29,30].

Existing Challenges	Proposed Approach
Challenge: Data ownership	
Existing client-server models might use personal information without owner’s consent and knowledge	in the blockchain model, every node can work as a server and does not need to rely on service providers to store personal information. Besides, data cannot be extracted without the network’s knowledge.
Challenge: Third party access	
Vulnerabilities in client server models makes it easy for the attacker to get access of sensitive information	in distributed structure of blockchain, data is distributed over a number of nodes, which removes the threat of data access via single entry point. Adversaries can get a portion of data that are cryptographically hashed and meaningless. Validating all transactions with the consensus of network nodes eliminates the need of third parties and records of transactions processed by the network be tampered with.
Challenge: Unauthorized access	
a vulnerability in a third-party service can grant access to all personal data beyond what the third party is storing.	Blockchains’ distributed structure and their strong cryptographic algorithms reduces the risk of unauthorized access to private data. An attacker with access to a validated third party can only access data where access was previously granted.
Challenge: Cost	
A drastic increase in the amount of IoT devices connected to cloud increased the need of computational capabilities	the distributed structure of the blockchain model and edge based computing architectures reduces the computational burden of individual nodes, including external cloud services.
Challenge: Data Manipulation	
Data manipulation risk is high for IoT external storages.	the blockchain model provides resilience to data compromise, as it cannot be forged because the information in the mined blocks is not allowed to alter.
Challenge: Server unavailability	
Centralized architectures that are based on cloud servers can fail if the connection to the server is broken.	the peer to peer nature of the blockchain-based transactions ensures that, if a connection between two nodes exists, then data can be transmitted independently of the availability of other nodes.

**Table 2:** Security Measures.

Parameter	Implementation
Authorization	Public key cryptography to encrypt CSK
Confidentiality	Public key cryptography and proof of authority
Integrity	Broadcast hash of each data_batch
Availability	Achieved by limiting number of requests
Anonymity	Discard raw data and store only processed information
